# Contribution of diminished kidney transplant GFR to increased circulating chemokine ligand 27 level

**DOI:** 10.1186/s12950-018-0194-7

**Published:** 2018-09-10

**Authors:** Ahmed Zahran, Ahmed Attia, Holly Mansell, Ahmed Shoker

**Affiliations:** 1 Nephrology Unit, Department of Medicine, Faculty of Medicine, University of Menoufia, Shibin El Kom, Egypt; 2 National Liver Institute, University of Menoufia, Shibin El Kom, Egypt; 3 College of Pharmacy and Nutrition, University of Saskatchewan, Saskatoon, SK Canada; 4 Department of Medicine, University of Saskatchewan, Saskatoon, SK Canada; 5 Saskatchewan Transplant Program, St Paul’s Hospital, 1702- 20th Street West, Saskatoon, SK S7M 0Z9 Canada

**Keywords:** Chemokines, Renal transplantation, Chemokine ligand 27 (CCL27), eGFR

## Abstract

**Background:**

Inflammatory chemokine ligands (CCLs) play an important role in cardiovascular disease and allograft injury. CCLs may independently associate with diminished estimated glomerular filtration rate (eGFR) in stable renal transplant recipients (RTR).

**Methods:**

Plasma levels of 19 CCLs (1, 2, 3, 4, 5, 8, 11, 13, 15, 17, 21, 24, 26, 27, CXCL5, 8, 10, 12 and 13) were measured in a cohort of 101 RTR. The cohort was divided according to CKD-EPI equation into three groups; group 1: eGFR ≥ 60 ml/min, group 2: eGFR 30–59.9 ml/min and group 3 eGFR ≤ 29.9 ml/min. ANOVA, Krusklwallis, Mann- Whitney Spearman correlation and regression analysis tests were used to determine association between reduced eGFR and inflammatory CCLs plasma levels measured by multiplex techniques. 20 healthy subjects with eGFR above 90 ml/min were included as control. Significance was sat at < 0.05.

**Results:**

Levels of CCLs 1, 4, 15, 27, CXCL8 and CXCL10 were significantly different among the four studied groups. Multivariate regression analysis (MVA) between eGFR and all CCLs demonstrated that CCL27 was the only ligand to remain significantly associated with diminished eGFR {*P* = 0.021 and *r* = − 0.35,(*P* = 0.001)}. In a second MVA between CCL 27 and patient’s demographics and laboratory variables, diminished eGFR, and elevated PTH, out of the twenty one available variables remained significantly associated with elevated CCL27levels.

**Conclusion:**

Diminished eGFR in stable RTR is associated with elevated plasma levels of CCL27. This association may explain, at least in part, the independent contribution of reduced eGFR to enhanced inflammation in RTR.

## Background

Chemokines and their ligands (CCLs) are a family of chemotactic cytokines that have ability to induce migration of different cell types especially those of lymphoid origin [1]. They coordinate leucocyte trafficking [2]. CCLs are expressed in tissues in response to injury or infection [3]. There are four families of chemokines; CCL, CXCL, CX3CL, and CL. Many chemokines can bind to multiple receptors and most receptors bind many chemokines [4].

The role of inflammatory CCLs in kidney dysfunction [5], including transplant injury [6], is widely recognized. These mediators play a pivotal role in acute allograft insult [7, 8] as well as chronic allograft injury and tissue fibrosis [9–11]. Plasma CCLs levels have been postulated to be biomarkers, which may assist in predicting transplant outcomes [12–17] as well as a therapeutic potential in kidney disease acute and chronic allograft nephropathy [18–20].

Chemokines were found to be associated with kidney function in diabetic kidney disease [21, 22]. Previously, we reported on the elevated plasma levels of CCLs in stable RTR [23].

The debate of the relative contribution of diminished eGFR to heightened inflammation in the renal patient remains unsettled.

We propose that the study of stable renal transplant patients with varying eGFR constitute a reasonable cohort to determine the contribution of diminished GFR to elevated plasma levels of CCLs, independent of other known factors. As such, the aim of this work was to characterize which circulating plasma chemokines ligands associated with diminished eGFR.

## Methods

This study included 101 stable renal transplant recipients and 20 healthy persons as a control group. The protocol for this study was approved by ethical committee of this institution. Informed consent was obtained from all participants. All RTR were older than 18 years with stable kidney function defined by no previous hospital admission and no change of serum creatinine more than 10% for the last 3 months. Patients with acute rejection, CMV disease or BK virus kidney disease diagnosed by a biopsy were excluded. All patients were followed at the Saskatchewan transplant program. Blood samples were collected for detection of CCLs. Multiplexed fluorescent bead-based immunoassay for detection of CCLs was described previously [24].

Nineteen CCLs (1, 2, 3, 4, 5, 8, 11, 13, 15, 17, 21, 24, 26, 27, CXCL5, 8, 10, 12 and 13) were measured.

RTR were divided arbitrary into three groups according eGFR, group 1: eGFR ≥ 60 ml/min, group 2: eGFR 30–59.9 ml/min and group 3: eGFR ≤ 29.9 ml/min.

The eGFR was calculated from the following equation:

GFR (CKD-EPI) =141X min(Scr/k,1)^α^ X max(Scr/k,1)^-1.209^ X.993^Age^ X1.018 [if female] X (1.159 [if black] where k = 0.7 if female, k = 0.9 if male, α = − 0.329 if female, α = − 0.411 if male, min = minimum of Scr/k or 1 and max = maximum Scr/k or 1, Scr = serum creatinine (mg/dL).

## Statistical analysis

Data were collected and analyzed using SPSS version 22 and excel sheet. Numerical data were presented as mean and standard deviation (SD) or median with 95% confidence interval as appropriate. Categorical data were presented as number and percentage. ANOVA test was used to compare normally distributed data between more than two numerical groups. For non-normalized data Krusklwallis test was used for comparing more than two groups while Mann-Whitney test was used for comparison between each two groups. Chi square test was used to compare groups with categorical data. Spearman correlation was used to test for association between variables. Univariate and multivariate linear regression analysis were used to detect the independent predictor of eGFR reduction in RTR. *P* value < 0.05 was considered significant.

## Results

This study included 101 stable RTR and 20 healthy persons as control group.. Nineteen CCLs were measured. Table 1 showed demographics and laboratory data of the stable RTR. Among the three patient groups, levels of hemoglobin, serum phosphorus, Parathyroid hormone, and urinary albumin creatinine ratio were significantly different. The rest of the twenty one demographic variables tested were similar including age and duration of transplantation.

**Table 1:** Demographic and laboratory characteristics in the studied patient groups

Variables	Group 1 eGFR. ≥ 60 (77.27 ± 17.07)	Group 2 eGFR 30–59.9 (46.69 ± 8.92)	Group 3 eGFR≤ 29.9 (21.43 ± 5.81)	ANOVA / K / Chi^2^	P
Age (Years)	47.04 + 15.14	52.10 + 12.45	50.15 + 15.04	1.28	0.28
Gender:					
M: No (%)	17 (54.8%)	25 (50%)	16 (80%)	5.4^a^	0.08
F: No (%)	14 (45.2%)	25 (50%)	4 (20%)		
BMI	27.23 + 4.27	29.01 + 5.43	27.32 + 5.76	1.5	0.23
Tx duration (Years)	7.48 + 5.75	6.31 + 4.20	8.95 + 3.88	2.4	0.09
SBP (mmHg)	128.3 + 12.7	127.1 + 14.0	134.7 + 18.3	2.0	0.14
DBP (mmHg)	77.65 + 8.55	79.1 + 11.4	79.3 + 9.9	0.23	0.8
Dialysis:					
HD	15 (57.7%)	22 (53.7%)	13 (72.2%)	1.8^a^	0.41
PD	11 (42.3%)	19 (48.3%)	5 (27.8%)		
Donor:					
Living	11 (39.3%)	20 (42.6%)	6 (30%)	0.93^a^	0.63
Deceased	17 (60.7%)	27 (57.4%)	14 (70%)		
Diabetes:					
Yes	9 (29%)	17 (34%)	6 (30%)	0.25^a^	0.88
No	22 (71%)	33 (66%)	14 (70%)		
Smoking:					
Yes	9 (29%)	8 (16.3%)	4 (20%)	1.90^a^	0.39
No	22 (71%)	41 (83.7%)	16 (80%)		
HB (g/L)	126.6 + 12.6	125.3 + 16.9	111.4 + 15.9	6.9	0.002
Ca (mmol/L)	2.36 + 0.13	2.35 + 0.17	2.34 + 0.27	0.04	0.95
PO4(mmol/L)	0.89 + 0.23	1.0 + 0.25	1.2 + 0.31	10.7	0.000
iPTH (pg/ml)	14.76 + 18.72	9.02 + 4.99	26.82 + 28.66	10.03^b^	0.007
Cholesterol (mmol/L)	4.47 + 0.93	4.20 + 1.30	4.98 + 1.16	1.8	0.17
TG (mmol/L)	1.75 + 0.72	2.03 + 1.14	2.19 + 1.14	1.2	0.3
LDL (mmol/L)	2.37 + 0.77	2.68 + 0.99	2.72 + 0.99	1.3	0.28
HDL (mmol/L)	1.31 + 0.44	1.41 + 0.44	1.24 + 0.39	1.3	0.29
Albumin (g/L)	37.37 + 4.34	38.59 + 3.65	35.90 + 5.31	2.9	0.06
ACR (mg/mmol)	26.14 + 33.81	78.9 + 123.2	186.5 + 163.6	17.8^b^	0.000
HS CRP (mg/L)	5.1 + 7.8	6.8 + 12.0	6.7 + 6.2	4.86^b^	0.088

*K* Krusklwallis, *eGFR* Estimated glomerular filtration rate *M* Male, *F* Female, *No* Number, *Tx* Transplantation, *SBP* Systolic blood pressure, *DBP* Diastolic blood pressure, *HD* Hemodialysis, *PD* Peritoneal dialysis, *HB* Hemoglobin, *Ca* Calcium, *PO4* Phosphorus, *iPTH* Intact Parathormone, *TG* Triglyceride, *LDL* Low density lipoprotein, *HDL* High density lipoprotein, *ACR* Albumin creatinine ratio, *HS CRP* High sensitive C reactive protein

^a^Chi square

^b^Krusklwallis

**Fig. 1:**
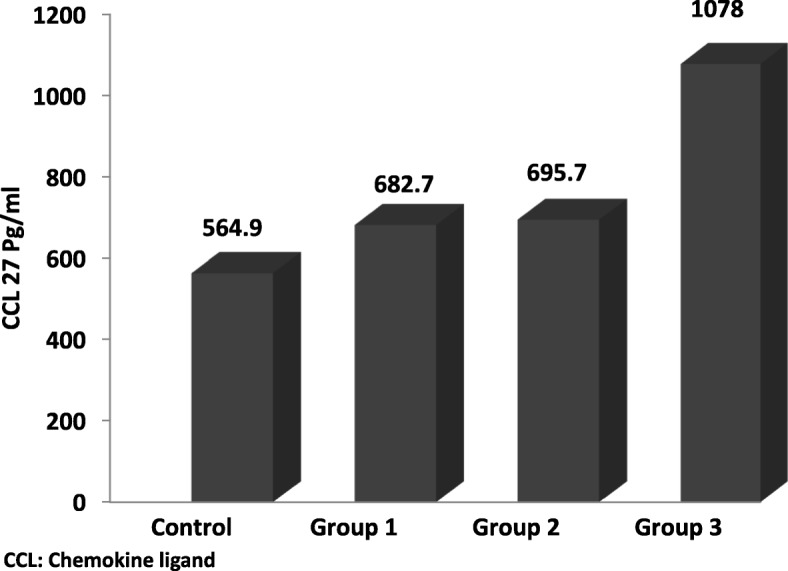
CCL27 levels in the studied groups

**Fig. 2:**
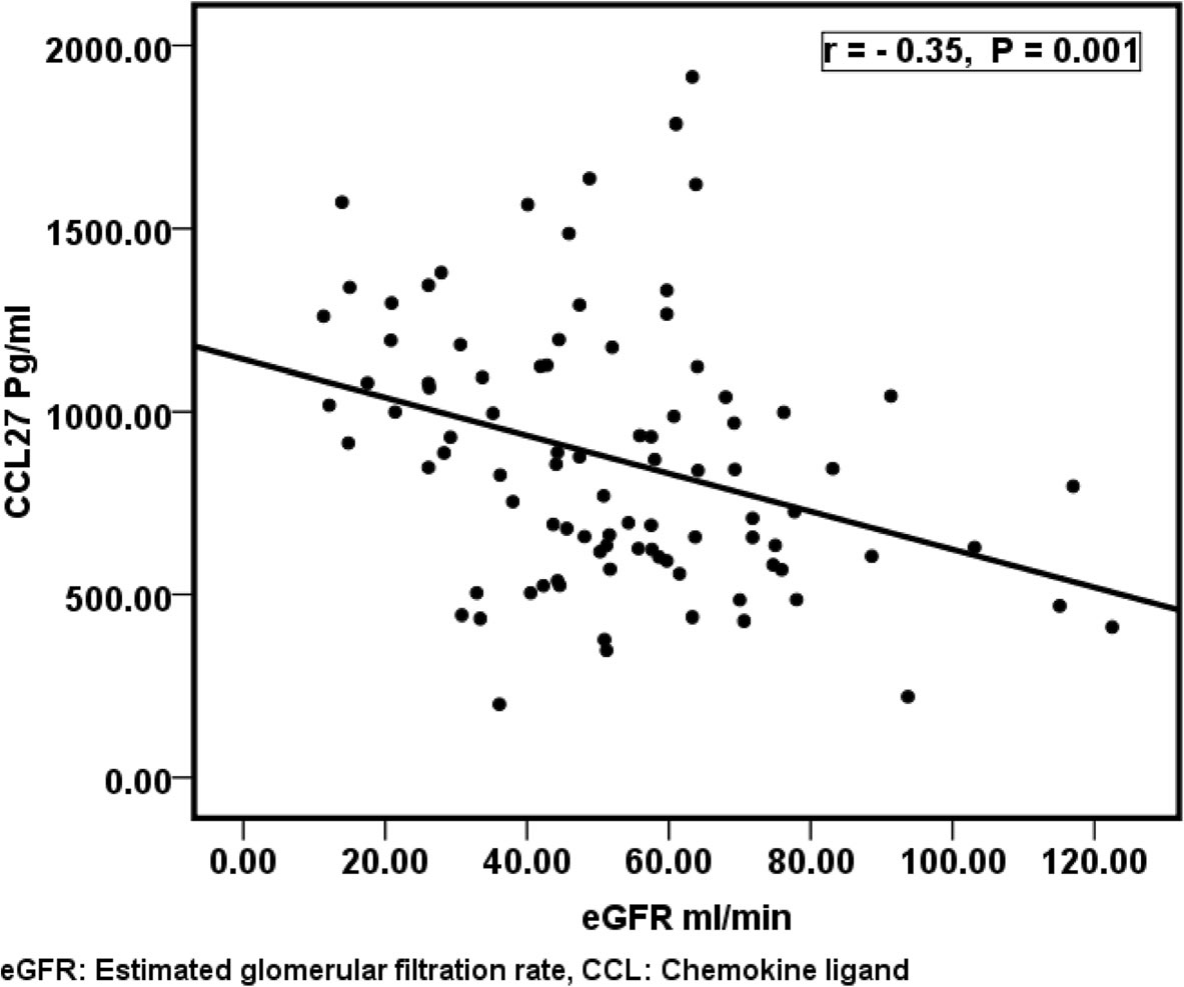
Spearman correlation between eGFR and CCL27 levels in renal transplant recipients

Table 2 shows comparison between the nineteen CCLs levels. All levels were presented as median and CI because of the wide variation in their respective levels and thus were non-normalized values. Comparison of studied groups showed that CCL1, CCL4, CCL15, CCL27, CXCL8 and CXCL10 were significantly different among the tested groups. Control CCL4, CCL15, CCL27, CXCL8 and CXCL10 levels were significantly lower than that in the three patient’s groups. Comparison between each two groups is further illustrated in Table 2. CCL27 was significantly elevated in group 3 compared to groups 1, 2 while there was no significant difference among groups 1 and 2 as seen in Fig. 1. Spearman correlation between eGFR and CCL27 showed significant negative correlation (Fig. 2).

**Table 2:** Comparison between CCLs in the studied groups

Chemokines Pg/ml	Control group (n 20)	Group 1 (n 31) eGFR. ≥ 60	Group 2 (n 50) eGFR 30–59.9	Group 3 (n 20) eGFR≤ 29.9	K	P	P1	P2	P3	P4	P5	P6
CCL1	1.95 (195–1.95)	1.95 (1.95–2.41)	1.95 (195–2.2)	2.9 (2.1–4)	15.8	0.001	NS	NS	0.000	NS	0.006	0.003
CCL2	140.26 (61.6–261)	105.4 (101.1–184.7)	140.5 (104. 3-178.5)	121.7 (100. 5-205.2)	0.2	NS						
CCL3	4.6 (2. 7-6.9)	4.17 (3. 2-5.5)	4.3 (3. 8-5.4)	3.9 (2. 9-5)	1.8	NS						
CCL4	25.8 (17. 3-55.4)	80.7 (22. 5-109.6)	63 (42. 8-93.5)	59.2 (31. 6-86.9)	11.9	0.008	0.028	0.000	0.014	NS	NS	NS
CCL5	6332 (3018–13,060)	5446 (3197–7945)	4189 (3225–6344)	2930 (1066–4812)	7.7	NS						
CCL8	20.53 (16. 17-26.19)	22.4 (18. 7-27.1)	26.3 (21. 3-31.2)	25.1 (18. 2-30.4)	3.9	NS						
CCL11	173.1 (25. 6-315.9)	111.8 (79. 4-176.5)	97 (39. 2-168.4)	60.6 (24. 5-88.7)	4.8	NS						
CCL13	43.96 (30-82)	49.2 (10. 7-69.6)	39.2 (20. 1-61.5)	38.4 (10. 7-94.4)	0.4	NS						
CCL15	2065 (1357–2798)	2725 (2208–3898)	3152 (2502–4246)	5769 (3738–7418)	20.6	0.000	0.011	0.003	0.000	NS	0.10	0.007
CCL17	21.34 (14.54–32.81)	21.7 (16. 1-29.6)	21.4 (17. 2-31.2)	37.7 (19. 9-51.4)	4.6	NS						
CCL21	29.03 (193. 4-392.6)	183.7 (100. 5-262.3)	182.7 (77. 4-262.3)	208.3 (77. 2-700.2)	2.1	NS						
CCL24	356.4 (176. 4-452.9)	262.7 (203. 9-347.9)	246.9 (188. 4-377.1)	328.3 (209. 7-487.9)	1.7	NS						
CCL26	48.83 (48. 8-49.8)	48.8 (48. 8-48.8)	48.8 (48. 8-48.8)	48.8 (48. 8-48.8)	6.8	NS						
CCL27	564.9 (444. 1-643.2)	682.7 (580. 8-844.5)	695.7 (625. 4-888.9)	1078 (930. 1-1339)	26.9	0.000	0.029	0.003	0.000	NS	0.000	0.001
CXCL5	419.3 (258. 1-574.5)	354.8 (255. 7-493.2)	402.2 (329. 5-518.2)	290.8 (159. 7-534.5)	2.3	NS						
CXCL8	3.99 (1. 7-7.8)	12.8 (8. 8-20.3)	11.7 (9. 7-15.2)	13.8 (7. 9-16.2)	18.9	0.000	0.000	0.000	0.000	NS	NS	NS
CXCL10	392.9 (281. 1-500.7)	930.7 (542. 5-1947)	1057 (749–1521)	739.3 (396. 2-1408)	19.4	0.000	0.000	0.000	0.003	NS	NS	NS
CXCL12	2027 (1446–3055)	1753 (1278–3102)	1603 (1136–2318)	2406 (990. 2-4122)	2.5	NS						
CXCL13	21.9 (14. 14-36.11)	31.9 (18. 2-82)	36.6 (27. 8-57.4)	37.2 (27. 8-77.5)	8.2	NS						

Levels of all chemokines expressed as median and 95% confidence interval

*CCL* Chemokine ligand, *eGFR* Estimated glomerular filtration rate, *K* Krusklwallis, *P1* Control versus Group 1, *P2* Control versus Group 2, *P3* Control versus Group 3, *P4* group 1 versus Group 2, *P5* Group 1 versus group 3, *P6* Group 2 versus Group 3, *NS* Non significant

Table 3 shows the univariate (UVA) and multivariate (MVA) analyses between the tested CCLs and eGFR. The UVA showed that CCL15 and CCL27 was independent associate with eGFR. While in the MVA, CCL27 was the only independent associate with reduced eGFR.

**Table 3:** Univariate and multivariate regression analyses of CCLs independently associated with reduced eGFR in transplant patients

Variables	Univariate regression		Multivariate regression	
	Standardized coefficient	*P* value	Standardized coefficient	*P* value
CCL1	−0.154	0.13		
CCL4	0.138	0.19		
CCL15	−0.275	0.008	−0.095	0.461
CCL27	−0.334	0.001	−0.300	0.021
CXCL8	0.089	0.43		
CXCL10	0.095	0.37		

*CCL* Chemokine ligand

### Correlative analysis between CCL27 levels and patient’s demographic and laboratory variables

We further, performed Spearman correlation, and multivariate regression analyses between CCL27 levels and the four demographic variables noticed to be significantly different among the patient groups in addition to eGFR. Table 4 showed significant correlation between CCL27 and hemoglobin, parathyroid hormone, and urinary albumin creatinine ratio and as expected eGFR. Multivariate regression analysis showed similar results while the MVA confirmed the significant association between elevated levels of CCL27 and reduced eGFR and to our surprise the significant relationship between elevated parathyroid hormone and CCL27 levels.

**Table 4:** Spearman correlation and multivariate regression analyses between CCL27 levels and patient demographics and laboratory variables

Variables	Correlation		Multivariate regression	
	r value	*P* value	Standardized coefficient	*P* value
HB (g/L)	- 0.30	0.005	−0.168	0.143
iPTH (pg/ml)	0.38	0.001	0.386	0.000
Phosphate (mg/dl	- 0.12	0.262		
ACR (mg/mmol)	0.21	0.045	0.043	0.671
eGFR (ml/min)	- 0.35	0.001	−0.227	0.039

*HB* Hemoglobin, *iPTH* intact parathyroid hormone, *ACR* Albumin creatinine ratio, *eGFR* Estimated glomerular filtration rate. The rest of all other demographic and laboratory variables were not significant

## Discussion

Chemokines are small hormone- like polypeptides which regulate cell trafficking and tissue homing [2]. Structure and biology of Chemokines and their ligands are well covered by extensive reviews [4].

The role of chemokines and cytokines in renal injury is well recognized [5] and others have investigated their roles in RTR [6–11].

Therefore we will limit our discussion to our positive results on the increased CCL 27 levels in the context of diminished GFR. First, our assay measures the human mature total CCL 27 and not any of its fragments as per the supplier communication.

Secondly, the increased sensitivity limits of the luminex immunoassay have made it possible to quantify extreme levels of circulating chemokines. The validity of these assay have been confirmed by other researchers [25, 26]. In our previous work we demonstrated elevated levels of most of the studied chemokines in stable RTR compared to control group [23] which may emphasize a state of generalized subclinical inflammation in apparently clinically stable patients. In our study we investigated the role of chemokines in context to eGFR in stable RTR.

It is recognized that CCL27 chemokine is a chemoattractant for cells bearing the CCR 10 receptor and which is found on keratinocytes [27], normal mucosa associated colon epithelium, trachea, mammary glands, retina and cells in the central nervous system [28].

It appears that T cell helper type 2 cytokines stimulate CCL27 secretion and CCL27 promote T helper type 1- activating antigen presenting cells’ It is suggested that CCL27 main function is to recruit lymphocytes to inflammatory tissues particularly the skin [29, 30].

CCL27 is also known as cutaneous T cell- attracting chemokine (CTACK) the only known receptor for CCL27 is CCR 10, which is expressed in normal skin and help to retain memory T cells homing during inflammatory skin conditions as well as immune surveillance [31].

In the skin, CCL27 is produced by keratinocytes and binds to CCR 10 receptor, characteristic of the majority of T lymphocytes infiltrating the skin. Specifically, CCL27 helps in lymphocyte adhesion in the skin, leading to pathological inflammatory skin diseases such as psoriasis, atopic dermatitis, mastocytosis [32] and drug induced cutaneous reactions [33]. It has been shown that CCL27 triggers resistance in myeloma cells [34]. Likewise, CCL27 is found in normal nasopharyngeal tissue samples particularly those individuals with seropositive Epstein- Bar virus capsid antigen- specific IgA level (VCA-IgA- positive) and is suggested as a plasma novel biomarker for nasopharyngeal carcinoma [35]. More recently, elevated CCL27 levels in patients with multiple sclerosis was observed. These investigators went further to suggest the Skin- Brain connection hypothesis in which the skin is proposed to be involved in MS pathogenesis [30, 36].

On the other hand CCL27 has been suggested to show a better prognosis in patients with cutaneous malignant melanoma [37].

It is not known why CCL27 is elevated in transplant patients with diminished eGFR. It is unlikely to support diminished clearance as a main cause of elevated levels of CCL27 because other CCLs would have been also retained. It is possible that elevated blood CCL27 level is a response to other inflammatory molecules associated with diminished renal function. After all, renal insufficiency is an inflammatory state [38]. Psychophysical stress may enhance CCL27 production [39].

With that in mind we foresee clinical significance of our findings for future studies. For example, it is intriguing to postulate that Skin changes associated with reduced GFR [40] may be attributed to increased CCL27. There is a link between psoriasis and reduction in kidney function [41, 42] representing another skin kidney connection. Of further interest, CCL27 was found to be highly associated with cutaneous psoriasis [32, 43–45]. Similarly, higher incidence of skin cancer seen in organ transplantation [46, 47] may be related to high levels of CCL27. In this context, it is intriguing to postulate the existence of a renal- skin axis similar to that between the skin- brain connections postulated by other researchers [30]. Of further interest, CCR 10, the receptor for CCL27 ligand, is expressed by the podocytes [48] and thus a role for CCL27 in podocytes function and injury in chronic allograft nephropathy is a potential hypothesis for future studies. As such, our finding raises more interesting possibilities such as a role of CCL27 in maintains alloreactive memory by supporting skin memory T cell population [49, 50]. If true the skin can be targeted for therapeutic manipulations of CCL27 to reduce inflammation in transplant and kidney diseases. Taken together we raise the intriguing potential of CCL27 as a potential putative marker of those patients who may have progressive decline in kidney function and that it may be a target to protect kidney function.

Elevated PTH is considered a putative uremic toxin in patients with chronic kidney disease. PTH is reported to associate with elevated inflammatory markers such as serum monocyte chemokine protein 1 [51]. Our literature review failed to find a relationship between CCL27 and PTH and thus we cannot speculate on the mechanism by which elevated PTH may enhance production of CCL27 at this stage.

There are limitations to our work. First the clinical relevance of elevated CCL27 needs confirmation by further studies including patients with native kidney diseases. We did not measure tissue levels of CCL27 or its receptor CCR 10. A prospective study of repeated measurement of chemokine and changes in GFR were not done. Finally the cause of decreased renal transplant function such as chronic allograft nephropathy versus other causes of reduced eGFR was not studied in detail. Of note, all our patients were at least stable at the time of study. In addition we stress the fact that there was no significant relationship between CCL27 levels and recipient age, or the duration of transplantation.

## Conclusions

We report on the increased inflammatory chemokine ligands in transplant patients with reduced glomerular filtration rate. In particular, we found elevated blood levels of CCL27 in stable RTR with reduced GFR and raises the possibility of a skin- kidney connection to maintain alloreactivity. Our finding raises the intriguing possibility of targeting the skin as a mean to inhibit CCL27 and thereby decrease renal inflammation in transplant patients and even in native kidney diseases. Future work should test the likelihood that CCL27 can serve as a biological marker for patients who are at excessive risk for progressive decline in kidney function and whether blocking CCL27 may retard progression to kidney failure.

## Abbreviations

CCLs: Chemokine (C-C motif) ligand
CXCLs: Chemokine (C-X-C motif) ligand
eGFR: Estimated Glomerular Filtration Rate
RTR: Renal Transplant Recipient
